# Reduced Genetic Load and Inbreeding in Reintroduced African Wild Dogs Reflect the Benefits of Admixture

**DOI:** 10.1111/mec.70424

**Published:** 2026-06-12

**Authors:** L. Tensen, X. Wang, J. Watermeyer, C. du Plessis

**Affiliations:** ^1^ Applied Zoology and Nature Conservation, Zoological Institute and Museum University of Greifswald Greifswald Germany; ^2^ Department of Zoology, Centre for Ecological Genomics and Wildlife Conservation University of Johannesburg Johannesburg South Africa; ^3^ Center for Evolutionary Hologenomics, Globe Institute University of Copenhagen Copenhagen Denmark; ^4^ African Wildlife Conservation Fund, Chishakwe Ranch, Savé Valley Conservancy Birchenough Bridge Zimbabwe; ^5^ Endangered Wildlife Trust Johannesburg South Africa

**Keywords:** conservation translocations, genetic load, genetic rescue, *Lycaon pictus*, population admixture

## Abstract

Conservation translocations have become important assets in saving African wild dogs ( *Lycaon pictus* ) from local extinction, which have declined drastically due to anthropogenic pressures. In South Africa, wild dogs were eradicated except for a small, isolated population remaining in Kruger National Park. Due to reintroductions into private reserves, the country now holds a viable metapopulation of over 150 individuals that is used as a donor to repopulate other countries in southern Africa. This Range Expansion Project allows a unique opportunity to quantify the genomic effect of founder events, population isolation and conservation translocations. For this purpose, we harvested 30 whole genomes of wild dogs from Kruger, private reserves and outside protected areas. Demographic reconstructions indicate that populations were historically large (10,000–40,000 individuals) but began declining gradually ~500–100 kya, coinciding with Mid‐Pleistocene climate shifts, followed by a sharp collapse ~2–0.8 kya. The Kruger population showed a substantially higher genetic load and a greater extent of runs of homozygosity (ROHs), indicating that its reduced genetic variation is driven primarily by inbreeding rather than demographic history. In contrast, reintroduced populations in private reserves exhibited the highest genetic diversity and the lowest genetic load. The short ROHs and close genetic affinity to an individual from Kenya support the view that wild dogs historically functioned as a largely panmictic species, and that reconnecting isolated populations can restore evolutionary potential. The absence of drift and relaxed selection implies that genetic resilience can be restored through population admixture, providing valuable guidance for managing threatened species.

## 1 Introduction

Habitat loss and fragmentation shape population dynamics by limiting resource availability, increasing isolation, and constraining demographic and genetic processes (Wiegand et al. 2005). Small and isolated wildlife populations are of particular conservation concern because inbreeding can expose recessive deleterious mutations that were previously masked in heterozygotes, leading to genetic disorders and reduced reproductive fitness (Charlesworth and Willis 2009; Agrawal and Whitlock 2012). Long runs of homozygosity (ROHs) have therefore emerged as strong indicators of inbreeding depression and are associated with reduced fitness and survival (Kyriazis et al. 2025). Although purifying selection can remove deleterious alleles from the genome given time (Dussex et al. 2023), its efficacy is limited in small populations where drift can randomly increase the frequency of some of the harmful variants (Peischl et al. 2015). Moreover, long ROHs result from recent inbreeding and contain genomic regions with fewer generations of recombination, which allows purifying selection less opportunity to eliminate strongly deleterious mutations (Parreira et al. 2025). To counteract these genetic challenges and restore reproductive success in isolated populations, conservation translocations have become increasingly common (Soorae 2025; Seddon and Redford 2025). When appropriately sourced from large and genetically diverse populations, such interventions can sustain long‐term genetic viability by introducing novel variation and reducing inbreeding, an outcome known as genetic rescue (Bell et al. 2019; Miller et al. 2020).

Conservation translocations have played a central role in preventing local extinction of the African wild dog ( *Lycaon pictus* ) (Davies and du Toit 2004; Davies‐Mostert et al. 2009), a species that has undergone severe declines in recent decades due to anthropogenic pressures (Woodroffe and Sillero‐Zubiri 2020). In South Africa, wild dogs were extirpated across most of their range, with only a single isolated population persisting in the fenced Kruger National Park (KNP). Subsequent reintroductions into private reserves have established a viable metapopulation (MTP) of over 150 individuals, using founders from KNP and free‐roaming populations in Botswana (Davies‐Mostert et al. 2015), which now serves as a donor source for reintroduction efforts across southern Africa (Tensen et al. 2022). In addition, free‐roaming (FRM) wild dogs have become reestablished in northern South Africa through natural dispersal from Zimbabwe and Botswana, occupying landscapes outside formal protected areas (Davies‐Mostert et al. 2012; Tensen et al. 2022). This FRM population experiences high levels of human–wildlife conflict and exhibits substantially lower genetic diversity than the MTP (Tensen et al. 2019). Understanding how genetic drift and range expansion shape these populations is essential for designing effective translocation strategies, including the selection of suitable individuals for establishing or augmenting satellite populations (Whiteley et al. 2015). Current management generally involves moving single‐sex groups and pairing them with unrelated partners prior to release, mimicking natural dispersal dynamics (Marneweck et al. 2019). Because the African wild dog Range Expansion Project is among the most extensive global reintroduction initiatives for an endangered carnivore (Spiering et al. 2011), it provides a unique opportunity to quantify the genomic consequences of founder events, population isolation and conservation translocations (Weimar et al. 2025).

Genome erosion and overall genomic health have become central themes in conservation efforts, particularly in species that have undergone severe bottlenecks or founder events (e.g., von Seth et al. 2022; Wang et al. 2023; Fontsere et al. 2024). In this study, we examine three African wild dog populations: the translocation‐connected metapopulation (MTP) established through reintroductions into private reserves; the fenced and demographically stochastic population in Kruger National Park (KNP); and the free‐roaming (FRM) population persisting outside protected areas. These populations have been separated for more than 10 generations, following the first reintroductions in the 1980s (Tensen et al. 2022), and each now exhibits a distinct genetic signature despite originating from the same founder stock and experiencing occasional admixture (Tensen et al. 2019; Weimar et al. 2025). Using the first whole‐genome comparison across these groups, we assess (i) how recent demographic history shapes spatial patterns of genetic diversity; (ii) the accumulation of deleterious alleles; and (iii) whether relaxed selection is associated with genomic variation at loci putatively linked to functional traits in reintroduced, isolated and naturally dispersing populations. Our overarching aim is to quantify how population size, isolation and translocation history have influenced genome‐wide diversity, genetic load and the direction of selection. Given the species' continuing fragmentation and its Endangered status under the most recent IUCN assessment (Woodroffe and Sillero‐Zubiri 2020), these insights provide timely guidance for managing genetic variation in threatened species.

## 2 Methods

### 2.1 Sampling

South Africa maintains a managed African wild dog metapopulation (MTP) of approximately 150 adults and yearlings distributed across private reserves (WAG‐SA 2025). For this study, blood samples were collected from multiple MTP reserves (*N* = 10) during translocation operations conducted between 2015 and 2023 (Table S1). Free‐roaming (FRM) wild dogs occur in northern South Africa, primarily in the Waterberg region and northern Limpopo. This population remains vulnerable due to high levels of human–wildlife conflict and typically fluctuates around 20 adults and yearlings (WAG‐SA 2025). A larger, more stable FRM population occurs in southern Zimbabwe, estimated at 70–90 individuals (AWCF 2025), and serves as the source of natural dispersers entering South Africa (Tensen et al. 2022). Blood samples from FRM individuals (*N* = 10) were collected in Waterberg and Limpopo (South Africa) and in the Savé Valley Conservancy (Zimbabwe) during de‐snaring operations between 2010 and 2015. In addition, 10 whole genomes from KNP were obtained from Meiring et al. (2022). Although the KNP population is unmanaged and demographically stochastic, it has remained relatively stable at approximately 125 adults and yearlings (WAG‐SA 2025).

### 2.2 Genome Sequencing

For laboratory processing, DNA was extracted using the QIAgen Blood & Tissue Kit (Qiagen, Valencia, CA, USA), following the manufacturer's supplementary protocol for blood & tissue samples, and DNA extractions were transferred to the Wildlife Genomics Laboratory at the University of Johannesburg. Double‐stranded DNA was quantified using Qubit, and integrity was assessed visually on agarose gels. Samples with molecular weight > 100 kb were enzymatically digested and randomly sheared to produce fragments with an approximate insert size of 330–530 bp. Fragmented DNA was 3′‐adenylated, ligated to sequencing adaptors, PCR‐amplified and purified. Library concentration and fragment‐size distributions were assessed prior to pooling. Sequencing was performed by a commercial provider on an Illumina HiSeq 2500 platform using paired‐end 2 × 150 bp chemistry targeting ~15× coverage. Raw reads were assessed for quality using FastQC v 0.11.9 (Andrews 2010). Adapters were removed and reads were quality‐trimmed with Fastp v0.23.4 (Chen et al. 2018), including 3′‐end trimming (Q ≥ 20) and removal of reads shorter than 50 bp. Filtered reads were aligned to the chromosomal African wild dog reference genome (GCA_040955705.1; Kliver et al. 2025) using BWA‐MEM v2.2.1 with default parameters (Li 2013). Resulting alignments were sorted with SAMtools v1.9 (Li et al. 2009), and duplicate reads were removed using Picard v2.9.1 (http://broadinstitute.github.io/picard/). Quality measures of sequencing alignment data stored in BAM files were retrieved with Qualimap v2.3 (García‐Alcalde et al. 2012).

### 2.3 Variant Calling

To meet the requirements of different downstream analyses, we generated two genotype datasets. Dataset 1 consisted of genotype likelihoods (GLs) estimated using ANGSD v0.940 (Korneliussen et al. 2014), which accommodates variation in sequencing depth among our samples and those from Meiring et al. (2022). GLs were computed with the GATK model (−gl 2; McKenna et al. 2010). Major and minor alleles were inferred directly from the GLs (−doMajorMinor 1), allele frequencies were estimated (‐doMaf 1). Only sites passing stringent SNP filtering criteria (SNP *p*‐value ≤ 2 × 10^−6^) were retained. Analyses were restricted to sites with base quality ≥ 20 and mapping quality ≥ 30. Additional filters included removal of low‐quality reads, minimum effective sample size (‐minInd 25) and minimum minor allele frequency (‐minMaf 0.05). Genotype likelihoods were exported in BEAGLE format for population structure analyses. Dataset 2 comprised called genotypes generated using BCFtools v1.9 (Danecek and McCarthy 2017), restricted to chromosomal scaffolds. Variant filtering was guided by summary statistics from VCFlib v1.0.3 (Garrison et al. 2022) and implemented using VCFtools v0.1.16 (Danecek et al. 2011). Filters included maximum missingness (‐‐max‐missing) 0.80, minor allele frequency (‐‐maf) 0.01, minimum variant quality (‐‐minQ) 20, minimum depth (‐‐minDP) 5 and maximum depth (‐‐maxDP) 50. Linkage pruning was performed with PLINK2 v1.90beta7 (Purcell et al. 2007). For analyses requiring an ancestral reference, we included one individual from Kenya (Armstrong et al. 2019), and the Ethiopian wolf ( *Canis simensis* ; SRR21985480; Mooney et al. 2023) was used as an outgroup. Ethiopian wolf ( *Canis simensis* ) and African wild dog ( *L. pictus* ) diverged approximately 3 million years ago (Gopalakrishnan et al. 2018; Chavez et al. 2019).

### 2.4 Population Structure

Using Dataset 1, we first explored population structure with a principal component analysis (PCA) implemented in PCAngsd v1.10 (Meisner and Albrechtsen 2018), specifying four eigenvectors in the iterative covariance estimation procedure. Pairwise Identity‐by‐State (IBS) distances were calculated in ANGSD by randomly sampling a single read per site and applying the strictref filter (‐doIBS), which computes allelic similarity (0 or 1) at positions with data for both individuals and averages across all sites. The resulting IBS matrix was used to construct a neighbour‐joining (NJ) tree with the R package APE v5 (Paradis and Schliep 2019). We estimated per‐individual admixture proportions using NGSadmix v32 (Skotte et al. 2013), running models from *K* = 2 to *K* = 5 with 10 independent replicates for each *K*. The maximum‐likelihood run was identified among converged replicates using EvalAdmix (Garcia‐Erill and Albrechtsen 2020). Genome‐wide genetic differentiation (pairwise *F*_ST_) between populations was calculated by estimating the joint site frequency spectrum (SFS) in realSFS v0.931 (Nielsen et al. 2012).

### 2.5 Demographic History

Historic effective population size (*N*_e_) was reconstructed using StairwayPlot v2.1.1 (Liu and Fu 2020) based on the folded site frequency spectrum generated with realSFS (ANGSD). Analyses were run with 500 bootstraps, exclusion of singletons, a sequence length of 4.9 Mb, 67% of sites used for model training and a mutation rate of 5.8 × 10^−9^ per site per generation (Campana et al. 2016). To infer population‐size trajectories deeper into the past, we applied the pairwise sequentially Markovian coalescent (PSMC) model (Li and Durbin 2011). Diploid consensus sequences were generated following recommended settings: BCFtools mpileup with ‐Q 10 ‐q 30, followed by vcf2fq with ‐d 5 ‐D 350 ‐Q 10 (Li 2011). PSMC was run using the parameters ‐N25 ‐t15 ‐r5 ‐p ‘4 + 25*2 + 4 + 6’. Results from both Stairway Plot and PSMC were scaled using a generation time of 4 years (O'Grady et al. 2008). Because Stairway Plot and PSMC are less reliable for contemporary *N*_e_ estimates (Nadachowska‐Brzyska et al. 2022), we additionally estimated recent *N*_e_ using the linkage‐disequilibrium method implemented in currentNe v1.0 (Santiago et al. 2024), reporting 90% confidence intervals. For this analysis, we used an unpruned Dataset 2 after removing alleles with > 20% missing data and retaining only those with minor allele frequency ≥ 0.05. In currentNe, we specified *k* = 1 to reflect a seasonal monogamous mating system, which best approximates African wild dog social structure (Asa and Valdespino 1998). Here, *N*_e_ represents the size of an idealised population whose rate of genetic drift matches that of the empirical population.

### 2.6 Heterozygosity

Using Dataset 2, we estimated nucleotide diversity (π) and absolute divergence (*d*_XY_) in non‐overlapping 10 kb windows across the genome using pixy v1.2.6 (Korunes and Samuk 2021), which incorporates invariant sites and accounts for missing data. Pixy was supplied with filtered all‐sites VCFs generated using *bcftools mpileup* and *bcftools call*, after removing indels, masking low‐quality genotypes (GQ < 20, DP < 5) and excluding sites with > 20% missing data. We estimated relatedness (r) using VCFtools v0.1.15 (Danecek et al. 2011), and calculated observed (*H*_O_) and expected (*H*_E_) heterozygosity with PLINK2. Runs of homozygosity (ROH) were identified on all autosomes using the *roh* function in BCFtools, using genotypes with a minimum quality threshold of ‐G 30. ROHs were classified as short (< 5 Mb), representing older demographic processes, or long (> 5 Mb), indicative of recent inbreeding. Individual inbreeding coefficients (*F*_ROH_) were calculated as the total length of all ROHs divided by the length of the autosomal genome covered by SNPs (2,384,003,008 bp). We also measured heterozygosity outside of ROHs by masking ROH regions (converted to BED coordinates) from the VCF and recalculating individual heterozygosity on the remaining sites using VCFtools (‐‐exclude‐bed, ‐‐het).

### 2.7 Genetic Load

To investigate the impact of genetic drift, we estimated per‐individual genetic load (i.e., deleterious mutations) using Dataset 1. Variant effects within coding sequences were annotated with Ensembl Variant Effect Predictor (VEP) v113.2 (McLaren et al. 2016), using the Kenyan wild dog genome (GCA_004216515.1; Armstrong et al. 2019) as the ancestral reference. Gene annotations from GCA_004216515.1 were transferred to our reference genome using LiftoffTools v1.6.3 (Shumate and Salzberg 2024). We focused on variants predicted as either high‐impact, loss‐of‐function (LoF) or moderate‐impact missense mutations, treating all such variants as putatively deleterious. Only biallelic single‐nucleotide variants with unambiguous ancestral alleles were retained, and sites heterozygous in the ancestral individual were excluded. For each individual, genotypes were coded relative to the ancestral allele as 0 (ancestral homozygous), 1 (heterozygous derived), or 2 (homozygous derived). Individual genetic load was summarised as counts of heterozygous and homozygous derived alleles, with population‐level summaries computed as sums or means across individuals. Variant‐level derived allele frequencies (DAF) within each population were calculated as the total number of derived alleles divided by the number of chromosomes genotyped. To assess the distribution of derived alleles across populations, variants were binned into frequency categories: 0–0.1, 0.1–0.5 and > 0.5. Differences in per‐individual cumulative genetic load among populations were tested using a one‐way ANOVA with population as a fixed factor with the R package ‘emmeans’ (Lenth 2021).

### 2.8 Selective Sweeps

To detect whether the populations were affected by divergent selective pressures, we performed cross‐population extended haplotype homozygosity (XP‐EHH) analyses (Simonson et al. 2010) for each SNP using R 3.6.3 (R Core Team 2024) and the ‘rehh’ package (Gautier and Vitalis 2012) with default parameters. XP‐EHH identifies hard selective sweeps by comparing haplotype homozygosity between two populations, with values strongly deviating from zero (> 2 or < −2) indicating a sweep in one population but not the other. We compared the MTP population against the two wild populations (FRM and KNP). Analyses were conducted on a nonpolarised dataset to retain variants with uncertain ancestral states, given the low genetic diversity of wild dogs, and without phasing SNPs (see Szpiech 2024 for phased vs. unphased comparisons). Median XP‐EHH values within nonoverlapping 50 kb windows were used to summarise regional haplotype signals.

In parallel, per‐site *F*_ST_ values between populations were calculated using VCFtools, retaining sites with minimum mapping quality ≥ 20 and base quality ≥ 20. Manhattan plots were generated to visualise genomic regions of high differentiation, with the 95th percentile of the empirical *F*_ST_ distribution used as a threshold for outlier SNPs. Outlier regions that significantly deviated from neutral expectations were interpreted as candidates for local adaptation via positive or balancing selection. Genes located in regions overlapping both high XP‐EHH and high‐*F*_ST_ signals were identified and functionally annotated using g:Profiler (https://biit.cs.ut.ee/gprofiler/gost). Gene functions were further curated with WebGestalt (Webgestalt.org) and manually verified through scientific literature and the NCBI database (www.ncbi.nlm.nih.gov) using the search terms ‘gene + function’.

## 3 Results

**FIGURE 1:**
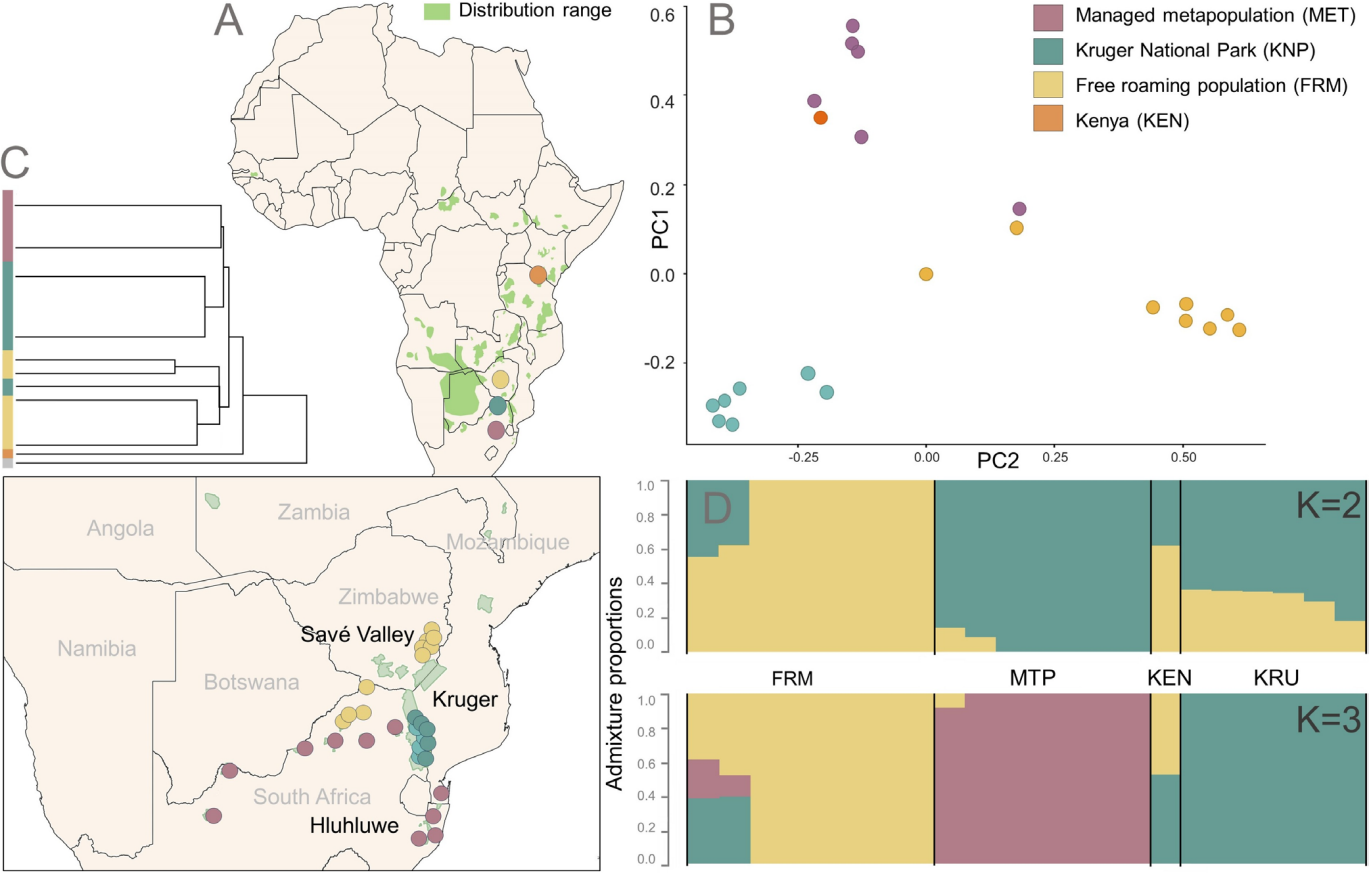
Population structure in African wild dogs. (A) Sampling localities in southern Africa, which are: the managed metapopulation (MTP) in private reserves, the free‐roaming population outside reserves (FRM) and Kruger National Park (KNP). A sample from Kenya was added as an ancestral group. (B) PCA of genetic variation in 31 individuals generated with PCAngsd, showing PC1 against PC2, which explained 12.84% and 8.50% of the total variation, respectively. (C) Neighbour‐Joining tree derived from the Identity‐By‐State (IBS) matrix. Samples are coloured by sampling locations. (D) Population ancestry proportions estimated in NGSadmix for *K* = 2 and *K* = 3.

We generated whole‐genome resequencing data for 20 African wild dogs sampled from the managed metapopulation (MTP) in South Africa and free‐roaming populations (FRM) in northern South Africa and southern Zimbabwe (Figure 1A; Table S1). The average sequencing depth for these individuals was 16.62× (SD = 251.83; Table S2). Sequences from Kruger National Park (KNP) were also included, with an average depth of 31.21× (SD = 8.10). To account for differences in sequencing depth, analyses based on genotype likelihoods in ANGSD were performed, including one additional individual from Kenya (East Africa). Mapping to the reference genome resulted in 234,866,416 genomic sites, of which 3,945,995 high‐quality sites were retained after filtering. Variant calling with BCFtools identified 2,402,099,369 SNPs, of which 2,068,868 passed strict filtering and were used for downstream analyses.

### 3.1 Population Structure

Population clustering was evident in the PCA (Figure 1B), with the first two components explaining 12.84% (PC1) and 8.5% (PC2) of the total genetic variation. Although the MTP population originated from reintroductions from KNP and FRM, MTP individuals exhibited closest genetic resemblance to the Kenyan individual. No clear structure was observed in PC3 (6.05%) or PC4 (5.26%) (Figure S1). A distance‐based neighbour‐joining (NJ) tree derived from the Identity‐By‐State (IBS) matrix showed generally long branches, indicating low differentiation among sampled wild dogs, including the Kenyan individual (Figure 1C). Nevertheless, MTP individuals formed a distinct cluster within the tree. Admixture analysis was used to further explore population structure. The most likely number of clusters was *K* = 3, based on estimated admixture proportions (Figure 1D) and the highest log‐likelihood values (Figure S2). Admixture plots revealed shared ancestry among FRM and Kenyan individuals. Pairwise residual correlations from EvalAdmix (Figure S3) further supported the presence of admixture and underlying population structure. To ensure that relatedness did not bias the results, we repeated the analysis after removing individuals (*N* = 8, FRM1, FRM2, KRU3, KRU6, KRU8, MTP1, MTP4, MTP8) with pairwise relatedness *r* > 0.15; this produced the same population structure (Figure S4). Genetic differentiation (*F*_ST_) was highest between FRM and KNP (0.075) and lowest between MTP and KNP (0.055). MTP was genetically most similar to the Kenyan individual, with an *F*_ST_ of 0.007.

### 3.2 Demographic History

**FIGURE 2:**
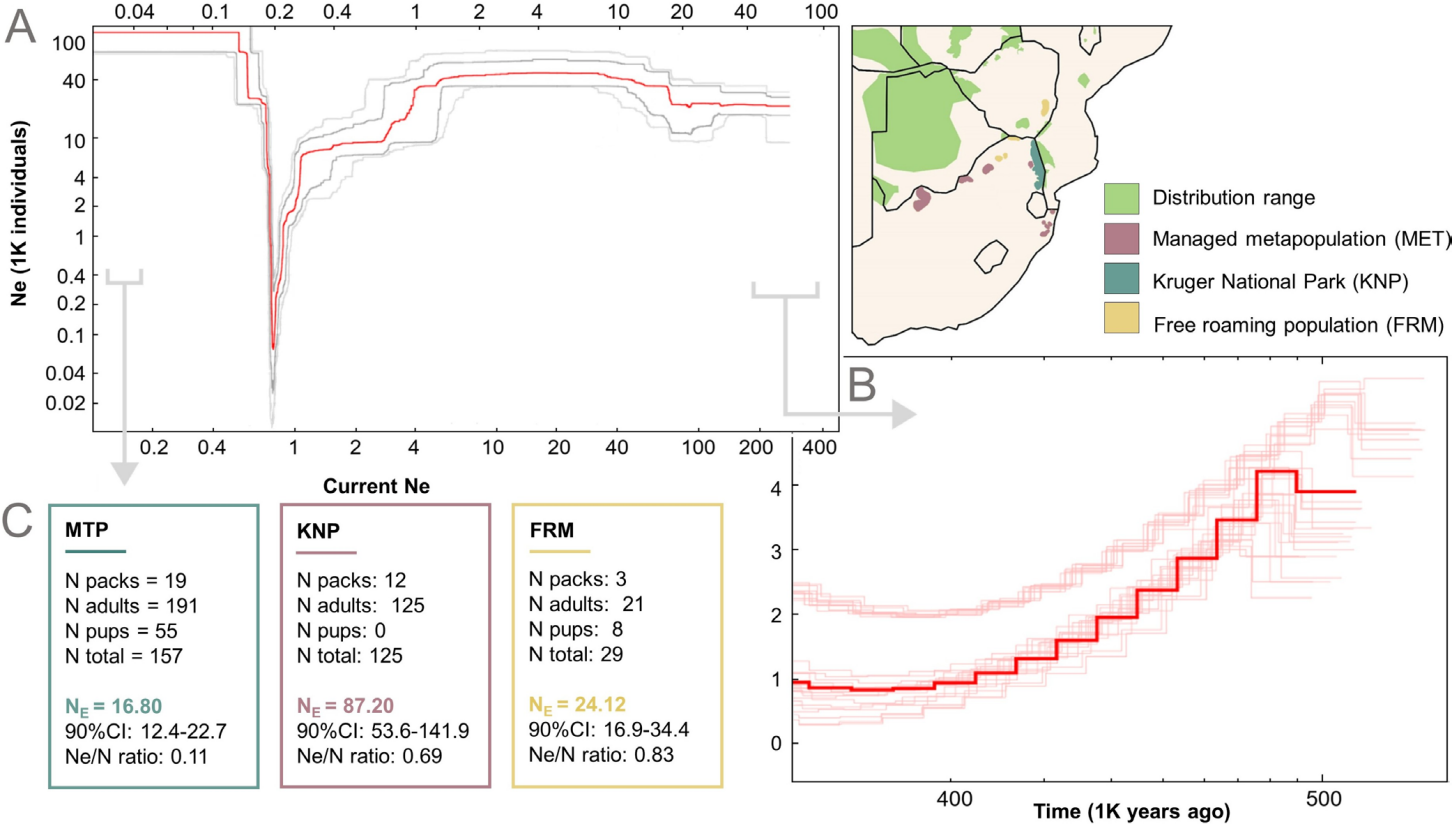
Demographic history of African wild dogs (A) Effective population sizes (*N*_e_) on a scale from 0 to 2 million years ago through a Stairway plot. Indicated is the Last Glacial maximum: 26.5 to 19 thousand years ago (kya). (B) Historical *N*_e_ reconstructed by using PSMC on a scale from 400 to 600 kya through assuming a mutation rate of 5.8 × 10^−9^ and a generation time of 4 years. (C) Effective population sizes (*N*_e_) measured with current *N*_e_ are indicated in boxes. Census size data is based on WAG‐SA reports (2025).

To reconstruct temporal changes in historical effective population size (*N*_e_), we applied StairwayPlot analysis. This revealed that regional African wild dog populations experienced expansions and contractions between ~2000–200,000 years ago (kya), followed by a pronounced population collapse around 0.8 kya (Figure 2A). Prior to the recent bottleneck, historic population sizes fluctuated between approximately 10,000 and 40,000 individuals. We also applied the pairwise sequentially Markovian coalescent (PSMC) to infer *N*_e_ further back in time (~500 kya). PSMC indicated a gradual population decline between 500 and 600 kya, followed by partial recovery beginning around 300 kya, consistent with the patterns inferred from Stairway Plot (Figure 2B). To estimate contemporary *N*_e_ and assess the effects of genetic drift, we used the linkage disequilibrium (LD) method. The MTP population showed an *N*_e_/*N* ratio of ~0.10, consistent with values typical of wild populations (Figure 2C). Estimates of current *N*_e_ were 16.80 (95% CI: 12.4–22.7) for MTP, 24.12 (16.9–34.4) for FRM and 87.20 (53.6–141.9) for KNP. Corresponding ratios of *N*_e_/*N* were 0.11 in MTP, 0.69 in KNP and 0.83 in FRM, highlighting pronounced differences in the strength of genetic drift among populations, with particularly strong drift potential in MTP relative to census size.

### 3.3 Genetic Diversity

**FIGURE 3:**
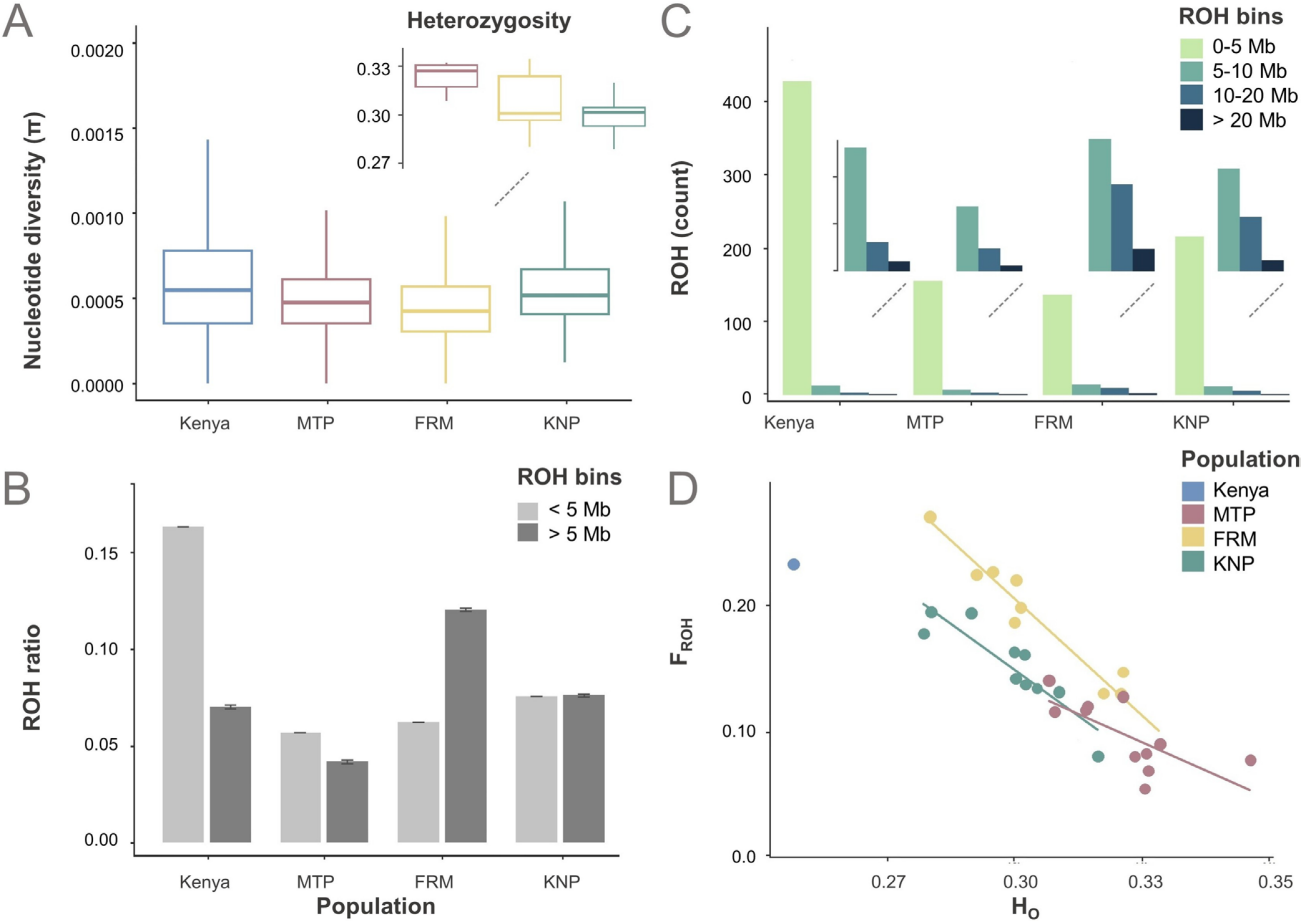
Genome‐wide diversity in African wild dogs, comparing the managed metapopulation (MTP), free‐roaming population (FRM) and Kruger National Park (KNP) in South Africa and Zimbabwe. (A) Nucleotide diversity measured with Pixy along the genome in non‐overlapping windows, and observed heterozygosity measured with PLINK2, where boxplots represent individuals pooled per population (*N* = 10 each). (B) Average count of runs of homozygosity (ROH) of different lengths. (C) The fraction of short > 5 Mb and long ROHs < 5 Mb of the total length of ROHs. Points in the boxplot are population means of per‐individual proportions (error bars are 95% CI of mean). (D) Correlation between observed heterozygosity (*H*_O_) and proportion of the autosomal genome in ROH (*F*_ROH_) comparing the three populations. *F*_ROH_ was calculated as the sum of ROH lengths divided by the length of the genome covered by the genome‐wide SNPs (2,384,003,008 bp).

To assess the impact of genetic drift on contemporary populations, we estimated genome‐wide nucleotide diversity (π) and heterozygosity using Dataset 2. Average nucleotide diversity was highest in MTP (0.0056 ± 0.0026), compared to FRM (0.00051 ± 0.00026) and KNP (0.00046 ± 0.00027), with differences significant across populations (ANOVA, F = 86.57, *p* < 0.0001; Figure 3A). Absolute divergence (*d*_XY_) averaged 0.0006 ± 0.0003 between populations, corresponding to roughly one nucleotide difference per 1700 bp. Observed heterozygosity (*H*_O_) was also highest in MTP (0.326 ± 0.014), compared to FRM (0.308 ± 0.018) and KNP (0.299 ± 0.013; ANOVA, F = 11.21, *p* < 0.0001; Figure 3A). To further explore homozygosity, we measured ROHs. Average ROHs were 117.3 ± 2.8 Mb in length in MTP (*N* = 139), 217.5 ± 3.7 Mb in FRM and 180.7 ± 2.8 Mb in KNP (Figure 3B). MTP displayed a higher proportion of short ROHs (< 5 Mb), whereas KNP had a greater proportion of long ROHs (> 5 Mb; Figure 3C), reflecting differences in recent versus older inbreeding and demographic history. The proportion of the autosomal genome in ROHs (*F*_ROH_) was lowest in MTP (0.098 ± 0.029), intermediate in KNP (0.152 ± 0.035) and highest in FRM (0.183 ± 0.056; ANOVA, F = 8.42, *p* = 0.0004). *F*_ROH_ and *H*_O_ were strongly negatively correlated (Spearman ρ = −0.84, *p* < 0.0001; Figure 3D), indicating that individuals with a higher burden of long homozygous tracts have reduced genome‐wide heterozygosity, consistent with recent inbreeding as a primary driver of genetic diversity loss. When ROHs were removed from the dataset and heterozygosity re‐estimated, the inter‐population differences disappeared, with mean *H*_O_ values of 0.72 in FRM and KNP, and 0.71 in MTP. This indicates that low genetic variation in wild dogs (especially in KNP) is driven primarily by inbreeding rather than long‐term demographic history. Patterns of reduced diversity and elevated inbreeding in the KNP were consistent across individuals (Table S3). Individual‐based estimates showed a relatively narrow range of inbreeding coefficients (*F*_ROH_ = 0.08–0.20) and heterozygosity (*H*_O_ = 0.28–0.32) in KNP, indicating that population‐level patterns are not driven by a small number of outliers. In contrast, MTP exhibited greater variability in both inbreeding (*F*_ROH_ = 0.09–0.27) and heterozygosity (*H*_O_ = 0.28–0.33), consistent with more heterogeneous genomic backgrounds.

### 3.4 Genetic Load

**FIGURE 4:**
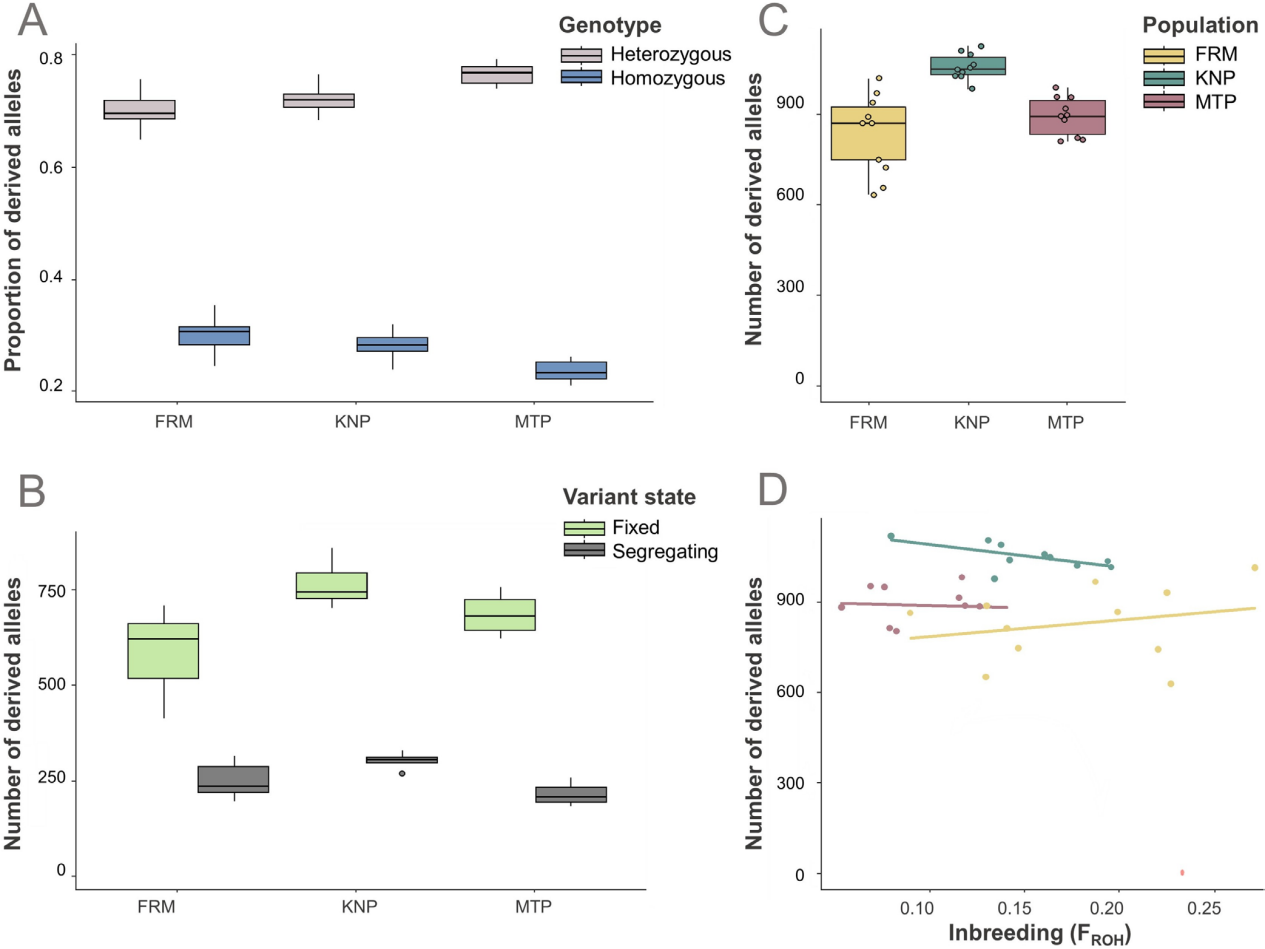
Genetic load (missense and loss‐of‐function mutations) in African wild dogs across three populations, comparing the managed metapopulation (MTP), free‐roaming population (FRM) and Kruger National Park (KNP) in South Africa and Zimbabwe (*N* = 10 per population). (A) Proportion of homozygous versus heterozygous derived alleles per individual. The lines in the boxplots are population means of per‐individual proportions (error bars are 95% CI of mean). (B) Fixed (homozygous) versus segregating (heterozygous) derived variants. (C) Per‐individual cumulative derived alleles. (D) Total derived allele counts were plotted against genomic inbreeding (*F*_ROH_) to examine the relationship between inbreeding and genetic load.

Genetic load across African wild dog populations was assessed by counting homozygous and heterozygous derived alleles for missense and loss‐of‐function mutations per individual. Homozygous load was highest in FRM, followed by KNP, whereas MTP exhibited the greatest heterozygous (i.e., masked) load (Figure 4A). The number of fixed (homozygous) derived alleles were most numerous in KNP (Figure 4B). Estimates of genetic load at the individual level revealed that KNP consistently exhibited higher numbers of homozygous derived alleles compared to individuals from reintroduced populations, indicating elevated realised genetic load (Table S4). MTP individuals carried more fixed derived alleles than FRM, while FRM had more segregating derived alleles than MTP (Figure 4B). Per‐individual cumulative genetic load (total derived alleles) was highest in KNP (ANOVA, *t* ratio = 5.74, *p* < 0.0001), with no significant difference between MTP and FRM (ANOVA, *t* ratio = −0.737, *p* < 0.468; Figure 4C). FRM showed the greatest variation among individuals, reflecting a broader distribution of homozygous and heterozygous derived alleles. Finally, the relationship between total derived alleles and genomic inbreeding (*F*_ROH_) differed among populations: KNP and MTP showed negative correlations, whereas FRM exhibited a positive correlation (Figure 4D). *F*_ROH_ values were lowest in MTP, widest in FRM and intermediate in KNP, suggesting that the interplay between inbreeding and genetic load varies across populations, likely reflecting differences in demographic history and population structure.

### 3.5 Selection

**FIGURE 5:**
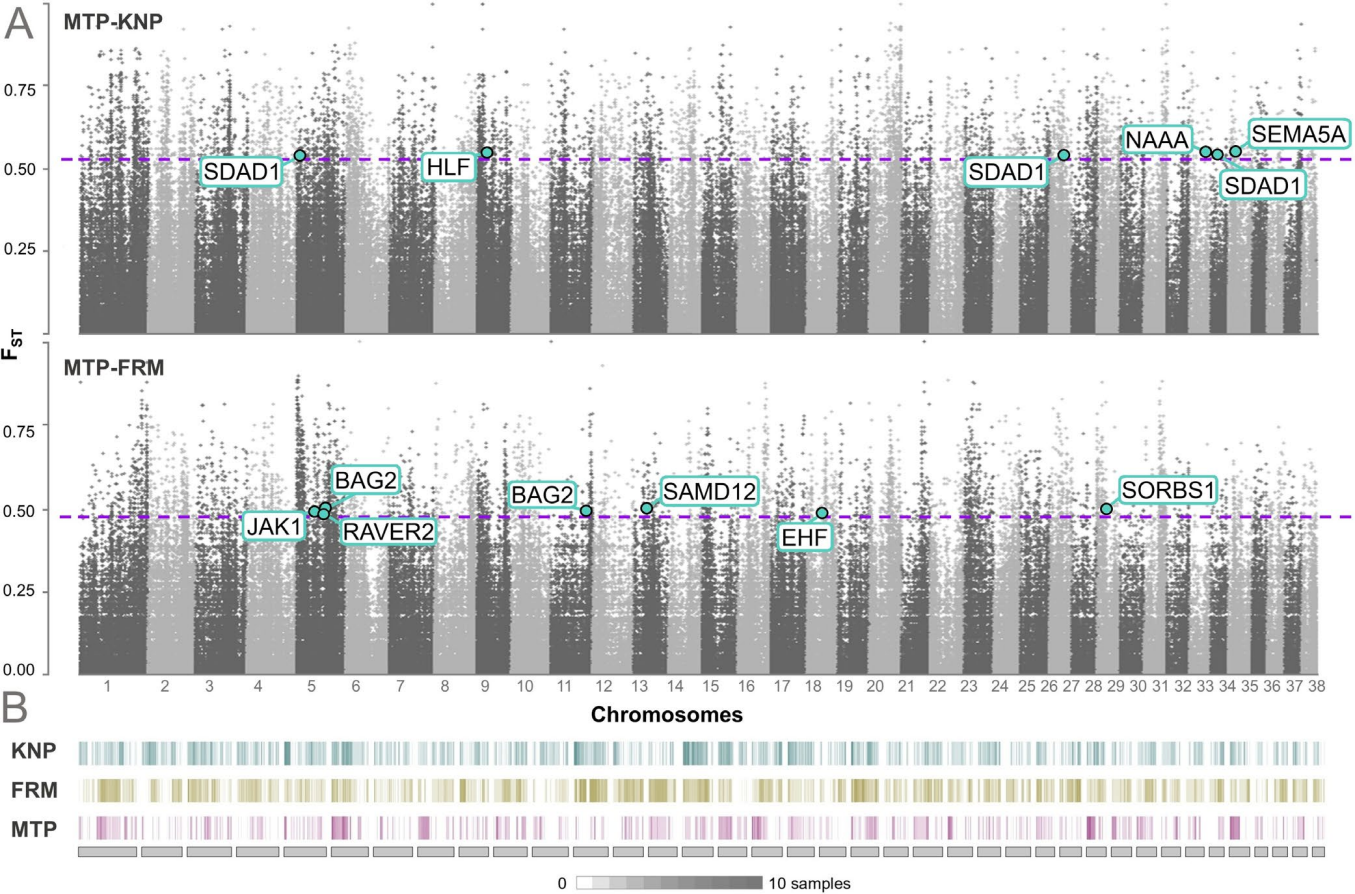
Candidate genes found to be under selection with XPEHH in African wild dogs. (A) Manhattan plot of per‐site *F*_ST_ across scaffolds. The purple dashed line indicates the 95th percentile of the empirical *F*_ST_ distribution. Genes under selection (XP‐EHH values < −2) are highlighted in blue. We specifically looked for genes that overlapped with *F*_ST_ outliers, outside runs of homozygosity (ROHs). (B) ROHs along the autosomal chromosomes. The darkness of the block refers to the number of the samples having a ROH in the region.

We investigated the presence of hard selective sweeps in FRM and KNP to assess whether selective signals may have been disrupted by admixture, potentially due to artificial pair bonding. We identified 4–16 genes potentially under positive selection in each population (Table S5). In comparisons between MTP and the wild populations (MTP–KNP and MTP–FRM), positive XP‐EHH values (exceeding > 2) indicate positive selection in MTP, whereas negative values indicate a sweep in the comparison population. All candidate regions identified in these comparisons showed strongly negative XP‐EHH values (< −2), indicating that selective sweeps are associated with the wild populations (FRM and KNP) rather than MTP. No enriched genes in KNP or FRM overlapped with windows of elevated *F*_ST_, that is, outlier SNPs. Some SNPs fell outside the 99% confidence interval, deviating significantly from expected heterozygosity, suggesting possible positive or balancing selection on adaptive traits (Figure 5A). However, gene ontology (GO) enrichment tests did not detect biologically informative categories. The most significant GO terms corresponded to root‐level categories, such as molecular function (GO:0003674), cellular process (GO:0009987) and biological_process (GO:0008150). A small subset of genes was associated with skeletal muscle development, RNA polymerase II transcription and DNA‐templated processes (Table S6). Overlaying *F*_ST_ outliers with ROH regions along chromosomes revealed that MTP had substantially less of the genome covered by ROHs longer than 100 kb in at least one individual (45%) compared to KNP (70%) and FRM (76%). These windows exhibited comparatively low *F*_ST_ values relative to the other populations, consistent with local inheritance from different source populations (Figure 5B).

## 4 Discussion

We analysed whole‐genome data for 30 African wild dogs to reconstruct their demographic histories and assess how inbreeding, drift and selection have shaped genomic variation in both reintroduced and free‐roaming populations. This represents a comprehensive genomic evaluation of the species, providing new insight into the genetic health of an IUCN‐listed endangered carnivore (Woodroffe and Sillero‐Zubiri 2020). Although the reintroduced populations ultimately trace back to Kruger National Park and free‐roaming wild dogs from South Africa and neighbouring regions, they nonetheless show subtle genetic differences in PCA and admixture analyses. These patterns likely reflect recent demographic processes, particularly elevated inbreeding compared to free‐roaming populations (signalled through higher cumulative genetic load), as well as genetic drift (Tensen et al. 2024). Consistent with this, FRM and KNP exhibit longer runs of homozygosity, suggesting increased vulnerability to inbreeding‐related fitness declines (Hasselgren et al. 2024). In contrast, the MTP population maintains high genetic diversity and higher masked load, facilitated by long‐term admixture among multiple founder lineages and repeated translocations (Dlugosch and Parker 2008; Lejeusne et al. 2014). Remarkably, given that wild dogs were first reintroduced into Hluhluwe‐iMfolozi Park in 1980, the absence of strong genomic signatures of drift or inbreeding in these reintroduced groups suggests that sustained gene flow and admixture may mitigate the long‐term genetic consequences of founder events. This underscores the power of outbreeding and sustained gene flow in maintaining genetic diversity and preserving adaptive potential.

Demographic analyses indicate that African wild dogs were once abundant in southern Africa, with effective population sizes fluctuating between ~10,000 and 40,000 individuals. A gradual decline occurred between ~500 and 100 thousand years ago (kya), coinciding with global climatic shifts toward cooler and drier conditions during the Mid‐Pleistocene (Deacon 1978). We suggest that these environmental changes, and the resulting alterations in faunal communities, contributed to reductions in wild dog population size (Bobe et al. 2002). Vegetation shifts during this unstable period, from tropical savannah to semi‐desert, caused many African mammals to become restricted to isolated savanna refugia, leaving strong genomic substructure in these species (Sithaldeen et al. 2015; Rocha et al. 2022). Interestingly, African wild dogs do not appear to follow this pattern. Population structure analyses place the East African genome close to southern African individuals, suggesting extensive historical connectivity. This may reflect the species' ecological flexibility: wild dogs inhabit semi‐arid ecosystems (Fanshawe et al. 1991) and can disperse over hundreds of kilometres (Davies‐Mostert et al. 2012), facilitating gene flow across large distances. Similarly, analyses of mitochondrial genomes (Tensen et al. 2025) and Major Histocompatibility Complex alleles (Weimar et al. 2025) indicate little divergence between eastern and southern African populations. Despite their connectivity, wild dogs still experienced significant demographic declines, likely due to their dependence on small‐ and medium‐sized, water‐dependent antelopes (Sandoval‐Serés et al. 2024). Drier conditions persisted until the early Holocene, when the climate shifted toward more mesic and tropical conditions (Martin 1968; Butzer 1974).

We detected a pronounced population bottleneck in African wild dogs approximately 2000–800 years ago. This period broadly overlaps with increasing human population densities, indicated by settlement expansion and larger farming areas (Loftus et al. 2025). The associated environmental changes in southern Africa may have contributed to wild dog population declines (Creel et al. 2020). During the 1900s, wild dogs were even systematically persecuted and classified as vermin, resulting in severe demographic losses (Childes 1988; Rasmussen 1999). The apparent sudden population recovery inferred by the Stairway Plot likely reflects limitations of site frequency spectrum (SFS)‐based demographic inference, which can be less accurate in recent time periods (Liu and Fu 2020). Contemporary estimates are primarily informed by rare variants, which are typically enriched among low‐frequency variants (Mathieson and McVean 2014). Notably, the MTP population exhibited the lowest contemporary effective population size (*N*_e_) despite relatively high heterozygosity. This pattern may reflect the small number of founders and ongoing subdivision and isolation among managed subpopulations, which can reduce *N*_e_ despite elevated genetic diversity (Caballero and Toro 2000; Gasca‐Pineda et al. 2013). In addition, we note that *N*_e_ estimates based on linkage disequilibrium may be sensitive to admixture, which can generate non‐equilibrium LD patterns and potentially bias *N*_e_ estimates in recently admixed populations (Waples and England 2011).

African wild dogs are cooperative breeders, with typically only an alpha pair reproducing (Spiering et al. 2010), which generally results in a reduced ratio of effective to census population size (*N*_e_/*N* ratio), a pattern also observed in other social carnivores (Lodé and Peltier 2005; Modi et al. 2021; vonHoldt et al. 2024). The *N*_e_/*N* ratio is a key diagnostic to illustrate the efficiency of genetic diversity retention (Wright 1931; Waples 2024). Very low *N*_e_/*N* ratios (< 0.05) can indicate high reproductive skew, uneven genetic contributions among breeders or recent severe bottlenecks (Hedrick 2005), which were not observed in wild dogs. Interestingly, the *N*_e_/*N* ratios observed in KNP (0.69) and FRM (0.83) are high compared to MTP (0.11) and unusually high for a cooperative breeder, likely reflecting specific demographic and genetic conditions (Frankham 1995; Hedrick 2005). In free‐ranging populations, subordinate females contribute approximately 10% of litters, a proportion that tends to be higher in small or isolated groups (Yordy and Mossotti 2016). Newly formed packs with closely related females also show significantly higher rates of multiple litters (Groom et al. 2024). The presence of related breeding females, such as litter sisters or mother‐daughter pairs, may reduce reproductive conflict, a pattern observed in other cooperative breeders like meerkats ( *Suricata suricatta* ) (Clutton‐Brock et al. 2001). Although recent work suggests that higher relatedness may promote multiple sires and influence reproductive skew (Groom et al. 2024), and that MTP may better resemble the natural state, further genetic analyses are needed to confirm this. Inbreeding avoidance likely also plays a key role, as limited access to unrelated mates has constrained population viability in both wild dogs (Becker et al. 2012) and meerkats (O'Riain et al. 2000). Such studies represent a promising avenue for understanding the interplay between cooperative breeding, genetic diversity and reproductive outcomes in wild dogs.

Runs of homozygosity are reliable markers of fitness losses through inbreeding, ithat is, inbreeding depression, as contiguous homozygous genomic stretches due to inheritance of identical haplotypes from related ancestors (Kyriazis et al. 2025). We found that these were present in FRM and KNP, and to a lesser extent in MTP. Likewise, *F*_ROH_ was found to be much lower in MTP. Long ROHs (> 1 Mb) tend to result from recent inbreeding between closely related individuals, whereas short ROH (< 1 Mb) are likely to have arisen from historical population bottlenecks (Ceballos et al. 2018; Shafer and Kardos 2025), since the length of ROHs is determined by the number of generations of recombination separating individuals since their common ancestor. Although we did not include fitness traits in this study, inbreeding depression (through lowered reproductive success) was previously detected in wild dogs (*F*_IS_ ≥ 0.25; Spiering et al. 2011), a level not observed in our dataset based on individual heterozygosity‐derived inbreeding coefficients (F). The highest F values were detected in FRM, as well as signals of outbreeding. Not surprisingly, this population originated from different founding populations through dispersal from Botswana and Zimbabwe (Tensen et al. 2022), but annually suffers severe demographic losses through illegal snares set for antelopes or targeted poisoning (Becker et al. 2013; Nicholson et al. 2020). High mortality rates are known to cause source‐sink dynamics and reduce effective dispersal across metapopulations (Jackson and Fahrig 2011). The KNP has been relatively small and isolated through the decades, but appears to avoid inbreeding within packs, thereby sustaining levels of heterozygosity (Meiring et al. 2022). Nonetheless, *F*_ROH_ was still over 30% higher in KNP compared to MTP (with 19% versus 14% of the genome covered in ROH), clearly illustrated the positive effect of outbreeding as a result of admixing different founder populations.

We assessed whether wild dog reintroductions increased genetic load through inbreeding and relaxed selection, as has been observed in other range‐expanding or recovering carnivore populations (Hagen et al. 2015; de Pedro et al. 2021), but found the opposite pattern. Both homozygous and heterozygous loads were highest in KNP, whereas FRM exhibited the greatest inter‐individual variation in the number of homozygous and heterozygous derived alleles. MTP showed comparatively lower overall genetic load, of which a larger proportion was masked in heterozygous form. Notably, patterns of inbreeding (*F*_ROH_), which reflect recent consanguinity, did not align with patterns of genetic load, which also capture the longer‐term accumulation of deleterious alleles. FRM appears to have undergone recent bottlenecks or founder events, generating high *F*_ROH_ through long runs of homozygosity; however, such demographic contractions can also facilitate efficient purging of recessive deleterious mutations, resulting in a lower overall burden of derived alleles (Robinson et al. 2023). Consistent with this, the elevated proportion of homozygous derived alleles in FRM, alongside a comparatively lower overall load of fixed deleterious variants, may indicate the early stages of purging. In contrast, MTP's managed breeding regime likely reduces inbreeding, lowering *F*_ROH_, yet allows mildly deleterious alleles to persist in heterozygous form, where they are largely shielded from selection and therefore slow to purge (Glémin 2003). These contrasting patterns illustrate that high homozygosity does not necessarily translate into elevated genetic load if purifying selection has already removed strongly deleterious variants, whereas populations with lower inbreeding can retain a higher segregating load.

Small populations can also experience shifts in selective pressures and altered fitness landscapes over time, potentially reducing their long‐term evolutionary potential (McPhee and McPhee 2012; Eizaguirre and Baltazar‐Soares 2014). Following reintroduction or natural re‐establishment, strong founder effects combined with genetic drift and inbreeding may initially elevate genetic load, although such deleterious variants are expected to be removed by purifying selection (Kyriazis et al. 2025). When founder events are followed by rapid demographic recovery, the overall loss of genomic diversity is often relatively small (Bertorelle et al. 2022), and the efficiency of both positive and negative selection becomes increasingly dependent on effective population size. In larger or quickly expanding populations, selection is more effective, whereas in smaller populations only strong selective forces leave detectable signatures (Bosse et al. 2019). Consistent with this expectation, we detected no evidence of differential or strong positive selection acting on wild dog alleles within the metapopulation, in contrast to signals observed in FRM and KNP.

The few candidate genes identified were related to skeletal muscle development, RNA polymerase II transcription, and DNA‐templated transcription, but these signals were weak and not clearly associated with demographic history or with potential consequences of artificial pair‐bonding or managed gene flow. As always, interpreting selection scans in low‐diversity populations requires caution, as modest signals may represent false positives (Lou et al. 2021). We were particularly interested in loci under selection that fell outside ROHs; notably, these windows exhibited relatively low *F*_ST_, compared with the other populations, suggesting that they were inherited locally from different source populations rather than arising through recent selective pressures (Peter 2016). Similarly, MTP appears to have inherited ROHs from both parental source populations, which have since begun to break down following admixture, as indicated by the density distributions. Finally, among all *F*_ST_ outliers (*F*_ST_ > 0.75), homozygosity was higher in FRM and KNP than in MTP, underscoring the beneficial effect of admixture in reducing homozygosity and potentially mitigating the accumulation of deleterious variants.

## 5 Conclusions

Together, our results show that even populations founded from relatively low‐diversity source populations, as reported for African wild dogs in previous genomic studies (Marsden et al. 2012; Tensen et al. 2022; Meiring et al. 2022), can regain diversity when admixture and connectivity are maintained. The genomic signatures across reintroduced wild dog populations, including higher heterozygosity, shorter ROHs, reduced genetic load and an absence of strong positive selection, demonstrate that demographic recovery and gene flow can effectively counteract the legacy of recent inbreeding. Far from accumulating mutational burden, these populations are shedding it, rebuilding genomic health within just a few generations. The deep historical declines we infer underscore the species' long‐standing vulnerability, yet the swift restoration of genetic resilience following reintroduction highlights its remarkable capacity to rebound when movement is facilitated. The tight genomic clustering between reintroduced groups and the Kenyan wild dog supports the idea that reconnecting isolated populations may partially restore historical patterns of gene flow (Seehausen 2004; Garant et al. 2007; Stronen et al. 2012).

The results are consistent with the potential benefits of admixing isolated or small populations, such as KNP, as a conservation strategy to sustain adaptive potential. Conservation translocations in wild dogs generally aim to balance increasing genetic diversity to reduce the effects of inbreeding and genetic drift in small populations, and maintaining evolutionary and phylogenetic distinctiveness among lineages (Weimar et al. 2025). In practice, emphasis is more often placed on alleviating inbreeding depression, as its fitness consequences are typically considered more immediate and severe than the potential risks of outbreeding depression (Frankham et al. 2011; Edmands 2007; Chan et al. 2019; Ralls et al. 2020). Our results are consistent with a low risk of outbreeding depression in South African wild dogs given the absence of strong signals of local adaptation, suggesting that gene flow via translocations or natural dispersal is likely to provide net genetic benefits relative to continued isolation (Linderoth et al. 2025). In line with IUCN guidelines (IUCN/SSC 2013), the use of multiple source populations in reinforcement or reintroduction programs may therefore be justified to maximise genetic diversity and improve long‐term population persistence under changing environmental conditions.

## Author Contributions

L.T. conceptualised the study. J.W. and C.P. collected samples. L.T. curated the data. L.T. and X.W. developed the methodology and conducted the investigation. L.T. performed the visualisation and wrote the original draft of the manuscript. L.T., X.W., J.W. and C.P. reviewed and edited the manuscript.

## Funding

This work was supported by the Deutsche Forschungs Geschaft (TE 1502/1‐1, project 493094679) granted to L. Tensen.

## Ethics Statement

This study has been approved in 2022 by the Endangered Wildlife Trust Ethics Committee (EWTEC2022_016). Samples were collected and transported in 2016 under permit 13/1/1/30/2/0‐2015/01/003889 and analysed at the University of Johannesburg, which was approved by the Faculty Ethics Committee under permit Tensen_201337943.

## Conflicts of Interest

The authors declare no conflicts of interest.

## Supporting information


**Figure S1:** Population clustering of three African wild dogs populations (managed metapopulation, MTP; Kruger National Park, KRU; free‐roaming, FRM) with a principal component analysis (PCA) implemented in PCAngsd. The first two components explained 12.84% (PC1) and 8.5% (PC2) of the total genetic variation, and the followed two components 6.05% (PC3) and 5.26% (PC4).
**Figure S2:**. Log‐likelihoods for cluster number *K* = 1–10 estimated by NGSadmix. for African wild dogs from South Africa and Zimbabwe, and one individual from Kenya.
**Figure S3:**. EvalAdmix (matrices) analysis for 30 African wild dog samples from southern Africa, exploring admixture proportions and the pairwise correlation of residuals for *K* = 2 to *K* = 5. The lower triangle in the EvalAdmix matrix shows the correlation of residuals between individuals, whereas the upper triangle shows the mean correlation within populations.
**Figure S4:**. Population admixture based on ancestry proportions measured with NGSadmix of African wild dogs from three African wild dog populations: the translocation‐connected metapopulation (MTP) established through reintroductions into private reserves; the fenced and demographically stochastic population in Kruger National Park (KNP); and the free‐roaming (FRM) population persisting outside protected areas. The number of discrete populations *K* = 2 to *K* = 5 are illustrated.
**Table S1:**. African wild dog samples, sequenced on an Illumina platform. Mitochondrial genomes were assembled. Full sequences were deposited in Genbank with unique Accession numbers. mtDNA = mitochondrial DNA, WGS = whole genome sequencing, MTP = managed metapopulation (in private game reserves), FRM = free‐KNP = Kruger National Park, acc. no = accession number.
**Table S2:**. Mapped summary statistics of whole genomes of African wild dogs from the South Africa and Zimbabwe. All sequences were mapped to the reference genome (GCA_040955705.1): Ind. = individual, No. = number, std. = standard, QC = quality control.
**Table S3:** Genetic diversity measures of 30 African wild dogs from South Africa and Zimbabwe: number of variant sites (*N* sites), observed heterozygosity (*H*_O_), expected heterozygosity (*H*_E_), inbreeding coefficient (F), number of runs of homozygosity (no. ROHs), total length in base pairs (bp) in ROHs (tot length ROHs) and the total length of the genome covered in ROH (*F*_ROH_).
**Table S4:** Genetic load measures of 30 African wild dogs from South Africa and Zimbabwe: number of heterozygous derived alleles (HET), homozygous derived alleles (HOM) and fixed and segregating derived alleles.
**Table S5:**. Genes potentially under positive selection in three wild dog populations (managed metapopulation—MTP, free‐roaming population—FRM and Kruger National Park—KNP) in South Africa and Zimbabwe, using XPEHH values. Genes overlap with windows with xpehh > 2 or < −2 and FST in 95 percentile.
**Table S6:**. Most significantly enriched GO terms in the tests were the root terms, for example, molecular function (GO:0003674), cellular process (GO:0009987), biological_process (GO:0008150) in Webgestalt.com.

## Data Availability

The datasets generated during the current study have been made available on NCBI Genbank under the project PRJNA1313134. The Kruger samples were available under project PRJEB47265.
