# Supplementary material for: Reduced Genetic Load and Inbreeding in Reintroduced African Wild Dogs Reflect the Benefits of Admixture
Source: Mol Ecol. 2026 Jun 12;35(12):e70424. doi: 10.1111/mec.70424 (PMC13261542; doi:10.1111/mec.70424)
Supplement: Supplementary file 1 — Figure S1: Population clustering of three African wild dogs populations (managed metapopulation, MTP; Kruger National Park, KRU; free‐roaming, FRM) with a principal component analysis (PCA) implemented in PCAngsd. The first two components explained 12.84% (PC1) and 8.5% (PC2) of the total genetic variation, and the followed two components 6.05% (PC3) and 5.26% (PC4). Figure S2:. Log‐likelihoods for cluster number K = 1–10 estimated by NGSadmix. for African wild dogs from South Africa and Zimbabwe, and one individual from Kenya. Figure S3:. EvalAdmix (matrices) analysis for 30 African wild dog samples from southern Africa, exploring admixture proportions and the pairwise correlation of residuals for K = 2 to K = 5. The lower triangle in the EvalAdmix matrix shows the correlation of residuals between individuals, whereas the upper triangle shows the mean correlation within populations. Figure S4:. Population admixture based on ancestry proportions measured with NGSadmix of African wild dogs from three African wild dog populations: the translocation‐connected metapopulation (MTP) established through reintroductions into private reserves; the fenced and demographically stochastic population in Kruger National Park (KNP); and the free‐roaming (FRM) population persisting outside protected areas. The number of discrete populations K = 2 to K = 5 are illustrated. Table S1:. African wild dog samples, sequenced on an Illumina platform. Mitochondrial genomes were assembled. Full sequences were deposited in Genbank with unique Accession numbers. mtDNA = mitochondrial DNA, WGS = whole genome sequencing, MTP = managed metapopulation (in private game reserves), FRM = free‐KNP = Kruger National Park, acc. no = accession number. Table S2:. Mapped summary statistics of whole genomes of African wild dogs from the South Africa and Zimbabwe. All sequences were mapped to the reference genome (GCA_040955705.1): Ind. = individual, No. = number, std. = standard, QC = quality control. Table [file MEC-35-e70424-s001.docx]

Supplementary Material

**Reduced genetic load and inbreeding in reintroduced African wild dogs reflect the benefits of admixture**

Tensen, L.^1,2^, Wang, X.^3^, Watermeyer, J.^4^, & du Plessis, C.^5^

1. Applied Zoology and Nature Conservation, University of Greifswald, Germany
2. Centre for Ecological Genomics and Wildlife Conservation, Department of Zoology, University of Johannesburg, Johannesburg, South Africa
3. Evolutionary Genetics Section, Globe Institute, University of Copenhagen, Copenhagen, Denmark.
4. African Wildlife Conservation Fund, Chishakwe Ranch, Zimbabwe
5. Endangered Wildlife Trust, Johannesburg, South Africa


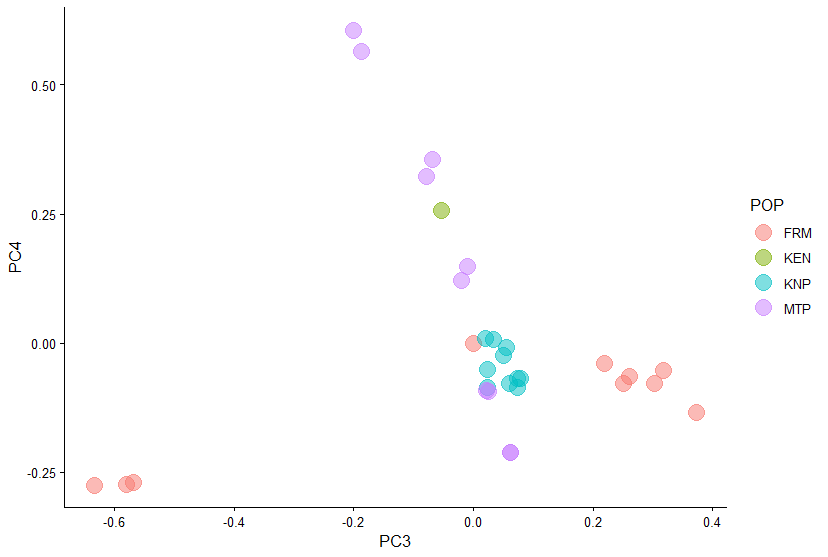


**Figure S1.**  Population clustering of three African wild dogs populations (managed metapopulation, MTP; Kruger National Park, KRU; free-roaming, FRM) with a principal component analysis (PCA) implemented in PCAngsd. The first two components explained 12.84% (PC1) and 8.5% (PC2) of the total genetic variation, and the followed two components 6.05% (PC3) and 5.26% (PC4).


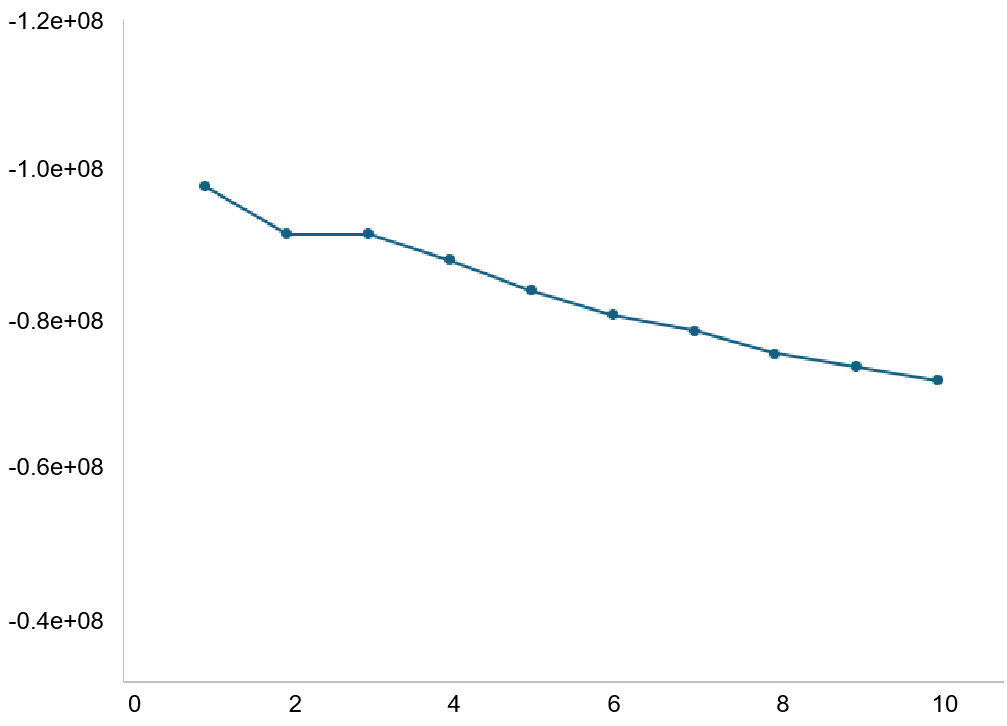


**Figure S2.** Log-likelihoods for cluster number K=1-10 estimated by NGSadmix. for African wild dogs from South Africa and Zimbabwe, and one individual from Kenya.


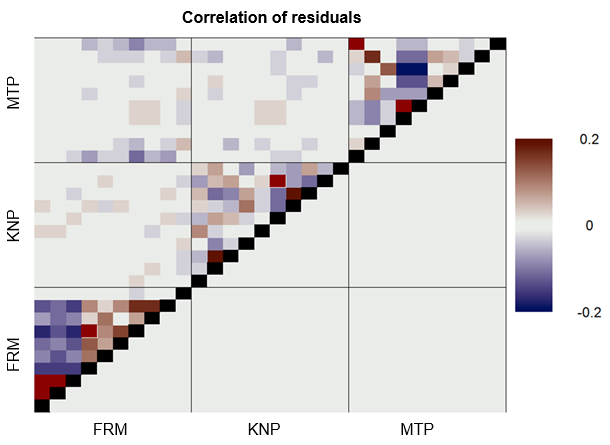


**Figure S3**. EvalAdmix (matrices) analysis for 30 African wild dog samples from southern Africa, exploring admixture proportions and the pairwise correlation of residuals for K=2 to K=5. The lower triangle in the EvalAdmix matrix shows the correlation of residuals between individuals, whereas the upper triangle shows the mean correlation within populations.


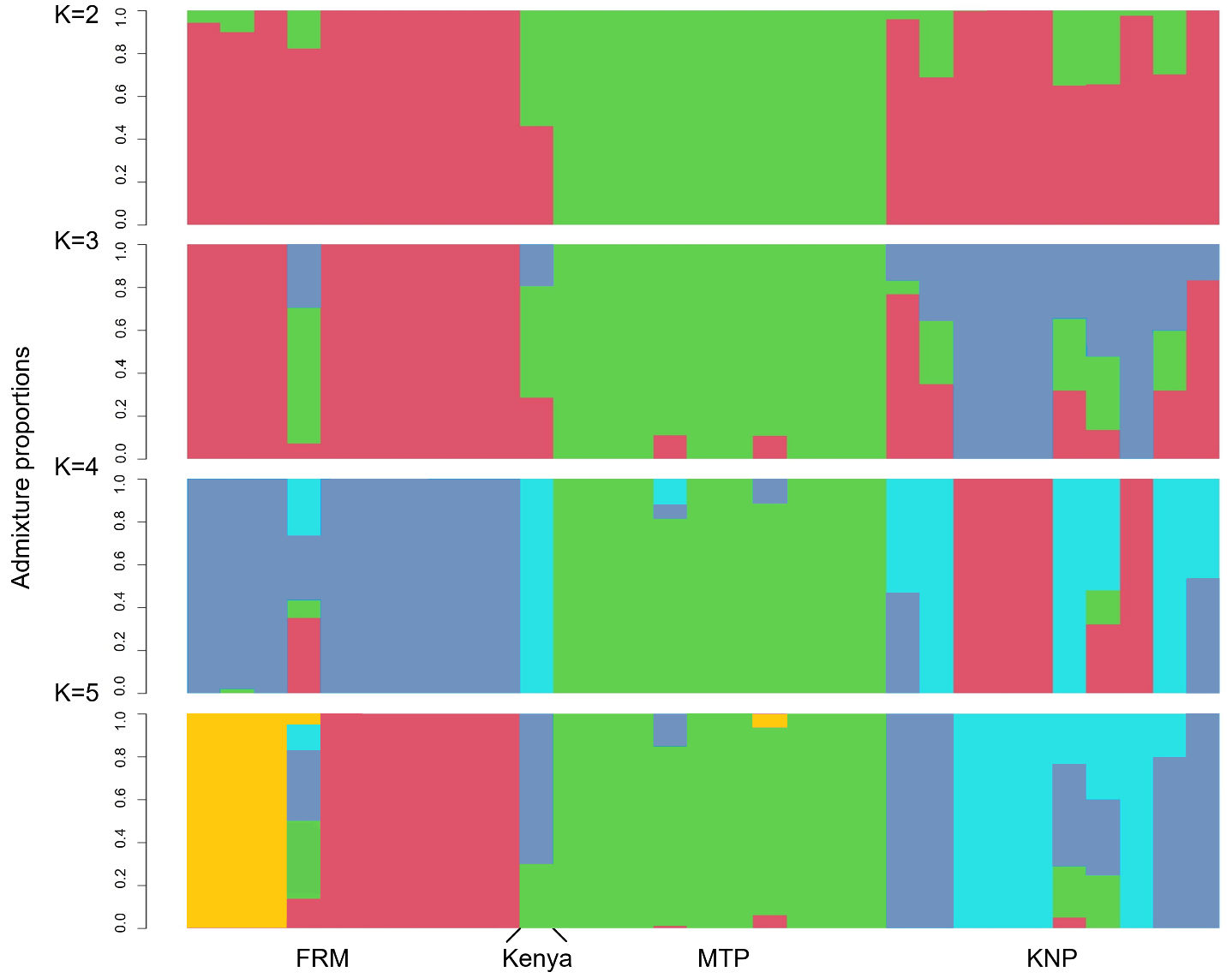


**Figure S4.** Population admixture based on ancestry proportions measured with NGSadmix of African wild dogs from three African wild dog populations: the translocation-connected metapopulation (MTP) established through reintroductions into private reserves; the fenced and demographically stochastic population in Kruger National Park (KNP); and the free-roaming (FRM) population persisting outside protected areas. The number of discrete populations K=2 to K=5 are illustrated.

**Table S1.** African wild dog samples, sequenced on an Illumina platform. Mitochondrial genomes were assembled. Full sequences were deposited in Genbank with unique Accession numbers. Abbreviations used: mtDNA = mitochondrial DNA, WGS = whole genome sequencing, MTP = managed metapopulation (in private game reserves), FRM = free-KNP = Kruger National Park, acc. no = accession number.

| Sample | Location | Location | Country | mtDNA acc. no. | WGS  acc. no. |
| --- | --- | --- | --- | --- | --- |
| FRM01 | Mapesu | Waterberg | South Africa | PV085278 | SRX30300051 |
| FRM02 | Twana | Waterberg | South Africa | PV085279 | SRX30300052 |
| FRM03 | Mogalakwena | Limpopo | South Africa | PV085280 | SRX30300053 |
| FRM04 | Lapalala | Limpopo | South Africa | PV085281 | SRX30300054 |
| FRM05 | Save Valley | Lowveld | Zimbabwe | PV085282 | SRX30300055 |
| FRM06 | Save Valley | Lowveld | Zimbabwe | PV085283 | SRX30300056 |
| FRM07 | Save Valley | Lowveld | Zimbabwe | PV085284 | SRX30300057 |
| FRM08 | Save Valley | Lowveld | Zimbabwe | PV085285 | SRX30300058 |
| FRM09 | Save Valley | Lowveld | Zimbabwe | PV085286 | SRX30300059 |
| FRM10 | Save Valley | Lowveld | Zimbabwe | PV085287 | SRX30300060 |
| MTP01 | Lapalala | Limpopo | South Africa | PV085268 | SRX30300050 |
| MTP02 | Manyoni | Mpumalanga | South Africa | PV085269 | SRX30300049 |
| MTP03 | Tembe | Kwazulu-Natal | South Africa | PV085270 | SRX30300061 |
| MTP04 | Pongolo | Kwazulu-Natal | South Africa | PV085271 | SRX30300062 |
| MTP05 | Madikwe | North West | South Africa | PV085272 | SRX30300063 |
| MTP06 | Tswalu | Kwazulu-Natal | South Africa | PV085273 | SRX30300064 |
| MTP07 | Hluhluwe | Kwazulu-Natal | South Africa | PV085274 | SRX30300065 |
| MTP08 | Mkuze | Kwazulu-Natal | South Africa | PV085275 | SRX30300066 |
| MTP09 | Khamab | North West | South Africa | PV085276 | SRX30300067 |
| MTP10 | Hluhluwe | Kwazulu-Natal | South Africa | PV085277 | SRX30300068 |
| KRU01 | Kingfisherspruit | Central | South Africa | - | ERR7672415 |
| KRU02 | Skukuza | South | South Africa | - | ERR7672418 |
| KRU03 | Imbali | Central | South Africa | - | ERR7672422 |
| KRU04 | Tshokwane | Central | South Africa | - | ERR7672424 |
| KRU05 | Crocodile bridge | South | South Africa | - | ERR7672445 |
| KRU06 | Kruger Gate | South | South Africa | - | ERR7672452 |
| KRU07 | Phalaborwa | North | South Africa | - | ERR7672455 |
| KRU08 | Malelane | South | South Africa | - | ERR7672462 |
| KRU09 | Sabi Sands | Central | South Africa | - | ERR7672469 |
| KRU10 | Shingwedzi | North | South Africa | - | ERR7672472 |

**Table S2.** Mapped summary statistics of whole genomes of African wild dogs from the South Africa and Zimbabwe. All sequences were mapped to the reference genome (GCA_040955705.1): Ind. = individual, No. = number, std = standard, QC = quality control.

| **Ind** | **No. reads** | **Mapped reads** | **Duplication rate** | **Mapping quality** | **QC %** | **Error rate** | **Mean coverage** | **std coverage** |
| --- | --- | --- | --- | --- | --- | --- | --- | --- |
| FRM01 | 203,542,926 | 99.76% | 39.82% | 53.29 | 41.68 | 0.0073 | 12.69 | 53.17 |
| FRM02 | 172,884,974 | 99.74% | 40.88% | 53.34 | 41.07 | 0.0071 | 10.77 | 35.14 |
| FRM03 | 232,007,840 | 99.75% | 41.02% | 53.21 | 42.23 | 0.0069 | 14.46 | 47.81 |
| FRM04 | 207,342,390 | 85.42% | 93.20% | 51.79 | 41.73 | 0.0053 | 11.07 | 21.93 |
| FRM05 | 189,932,384 | 99.78% | 41.82% | 53.37 | 41.82 | 0.0053 | 11.85 | 47.43 |
| FRM06 | 220,342,314 | 99.62% | 42.61% | 53.35 | 41.42 | 0.0057 | 13.72 | 60.12 |
| FRM07 | 189,932,384 | 99.78% | 41.82% | 53.37 | 41.14 | 0.0053 | 11.85 | 46.43 |
| FRM08 | 202,688,340 | 99.60% | 36.38% | 53.44 | 42.52 | 0.0056 | 12.62 | 62.35 |
| FRM09 | 225,949,716 | 99.27% | 41.91% | 53.14 | 41.61 | 0.0056 | 14.02 | 87.94 |
| FRM10 | 209,498,336 | 99.59% | 38.93% | 53.28 | 42.01 | 0.0051 | 13.03 | 99.34 |
| KRU01 | 443,034,810 | 99.65% | 11.33% | 53.33 | 41.15 | 0.0062 | 27.78 | 11.95 |
| KRU02 | 440,612,112 | 99.90% | 11.07% | 53.42 | 40.43 | 0.005 | 27.59 | 13.1 |
| KRU03 | 458,285,764 | 99.93% | 11.54% | 53.53 | 40.56 | 0.0047 | 28.71 | 13.26 |
| KRU04 | 431,487,422 | 99.55% | 11.02% | 53.33 | 40.91 | 0.0105 | 26.82 | 10.76 |
| KRU05 | 488,901,774 | 99.83% | 12.30% | 53.58 | 40.39 | 0.0046 | 30.61 | 14.69 |
| KRU06 | 457,498,702 | 99.88% | 11.88% | 53.51 | 40.48 | 0.006 | 28.63 | 11.88 |
| KRU07 | 367,465,656 | 99.89% | 13.20% | 53.16 | 44.21 | 0.007 | 22.97 | 17.42 |
| KRU08 | 384,797,136 | 99.18% | 11.63% | 53.57 | 39.84 | 0.0065 | 23.89 | 10.99 |
| KRU09 | 339,613,840 | 99.81% | 8.59% | 53.41 | 40.83 | 0.0065 | 21.23 | 10.51 |
| KRU10 | 344,151,120 | 99.85% | 8.60% | 53.46 | 41.26 | 0.0063 | 21.51 | 9.98 |
| MTP01 | 203,542,926 | 99.76% | 39.82% | 53.28 | 41.68 | 0.0073 | 12.69 | 53.17 |
| MTP02 | 243,444,630 | 99.60% | 47.54% | 53.16 | 41.35 | 0.0075 | 15.16 | 63.3 |
| MTP03 | 234,518,134 | 96.49% | 48.62% | 53.17 | 41.41 | 0.0076 | 14.12 | 60.91 |
| MTP04 | 230,054,926 | 97.42% | 44.65% | 52.96 | 42.34 | 0.0075 | 14 | 80.04 |
| MTP05 | 199,553,206 | 99.62% | 31.69% | 53.07 | 41.78 | 0.0067 | 12.42 | 55.72 |
| MTP06 | 245,807,286 | 99.68% | 48.53% | 53.25 | 41.48 | 0.0076 | 15.31 | 80.43 |
| MTP07 | 226,706,552 | 99.62% | 43.17% | 53.19 | 41.29 | 0.007 | 14.12 | 64.35 |
| MTP08 | 176,840,952 | 99.32% | 42.26% | 53.21 | 41.98 | 0.0054 | 10.98 | 40.72 |
| MTP09 | 239,940,644 | 97.84% | 47.37% | 53.43 | 41.03 | 0.0056 | 14.68 | 69.37 |
| MTP10 | 199,056,036 | 99.55% | 43.93% | 53.37 | 41.55 | 0.0054 | 12.39 | 43.53 |

**Table S3.** Genetic diversity measures of 30 African wild dogs from South Africa and Zimbabwe: number of variant sites (N sites), observed heterozygosity (H_O_), expected heterozygosity (H_E_), inbreeding coefficient (F), number of runs of homozygosity (no. ROHs), total length in base pairs (bp) in ROHs (tot length ROHs), and the total length of the genome covered in ROH (F_ROH_).

| INDV | N sites | H_E_ | H_O_ | F | No. ROHs | tot length ROHs | F_ROH_ |
| --- | --- | --- | --- | --- | --- | --- | --- |
| FRM01 | 456696 | 0.3014 | 0.3343 | -0.109 | 148 | 214897518 | 0.09 |
| FRM02 | 340897 | 0.3034 | 0.3211 | -0.0584 | 131 | 310169241 | 0.13 |
| FRM03 | 509650 | 0.3008 | 0.3001 | 0.0024 | 198 | 445132774 | 0.187 |
| FRM04 | 387805 | 0.3025 | 0.3258 | -0.0767 | 142 | 349764636 | 0.147 |
| FRM05 | 369415 | 0.3025 | 0.295 | 0.0249 | 148 | 540024719 | 0.227 |
| FRM06 | 463149 | 0.3014 | 0.3017 | -0.001 | 173 | 473295289 | 0.199 |
| FRM07 | 389476 | 0.3028 | 0.3005 | 0.0077 | 152 | 524495991 | 0.22 |
| FRM08 | 503815 | 0.3006 | 0.2803 | 0.0677 | 189 | 645901640 | 0.271 |
| FRM09 | 463454 | 0.3016 | 0.3251 | -0.0779 | 155 | 310172397 | 0.13 |
| FRM10 | 475502 | 0.3011 | 0.2913 | 0.0327 | 185 | 535970072 | 0.225 |
| KRU01 | 565470 | 0.2995 | 0.2787 | 0.0696 | 271 | 423651077 | 0.178 |
| KRU02 | 564722 | 0.2996 | 0.29 | 0.0323 | 222 | 463538417 | 0.194 |
| KRU03 | 566245 | 0.2999 | 0.3105 | -0.0353 | 228 | 312577756 | 0.131 |
| KRU04 | 565337 | 0.2996 | 0.3027 | -0.0102 | 253 | 328428659 | 0.138 |
| KRU05 | 564617 | 0.2997 | 0.3004 | -0.0025 | 243 | 338937396 | 0.142 |
| KRU06 | 563898 | 0.2994 | 0.2806 | 0.0628 | 221 | 465610962 | 0.195 |
| KRU07 | 563496 | 0.2995 | 0.3196 | -0.0673 | 195 | 190131242 | 0.08 |
| KRU08 | 563127 | 0.3002 | 0.3055 | -0.0176 | 243 | 319231383 | 0.134 |
| KRU09 | 568239 | 0.3003 | 0.3024 | -0.0069 | 223 | 384989585 | 0.161 |
| KRU10 | 568551 | 0.3002 | 0.3001 | 0.0001 | 233 | 387554947 | 0.163 |
| Kenya | 474221 | 0.3005 | 0.248 | 0.1744 | 445 | 556449154 | 0.233 |
| MTP01 | 440851 | 0.3018 | 0.3285 | -0.0886 | 148 | 190621804 | 0.08 |
| MTP02 | 468397 | 0.3011 | 0.3097 | -0.0283 | 199 | 277223179 | 0.116 |
| MTP03 | 437421 | 0.3013 | 0.3256 | -0.0807 | 161 | 305982628 | 0.128 |
| MTP04 | 492410 | 0.3006 | 0.317 | -0.0543 | 204 | 279847048 | 0.117 |
| MTP05 | 491656 | 0.3009 | 0.3173 | -0.0546 | 200 | 283490750 | 0.119 |
| MTP06 | 472094 | 0.301 | 0.3317 | -0.1017 | 174 | 163750380 | 0.069 |
| MTP07 | 456732 | 0.3016 | 0.3555 | -0.1786 | 121 | 184390133 | 0.077 |
| MTP08 | 393028 | 0.3018 | 0.3083 | -0.0218 | 153 | 336891241 | 0.141 |
| MTP09 | 445079 | 0.3017 | 0.3308 | -0.0963 | 158 | 129472976 | 0.054 |
| MTP10 | 418585 | 0.302 | 0.3311 | -0.0961 | 137 | 194312825 | 0.082 |

**Table S4.** Genetic load measures of 30 African wild dogs from South Africa and Zimbabwe: number of heterozygous derived alleles (HET), homozygous derived alleles (HOM), and fixed and segregating derived alleles.

| Individual | POP | HET | HOM | Total | Fixed | Segregating | Total |
| --- | --- | --- | --- | --- | --- | --- | --- |
| FRM01 | FRM | 555 | 602 | 1759 | 212 | 655 | 867 |
| FRM02 | FRM | 388 | 420 | 1228 | 192 | 462 | 654 |
| FRM03 | FRM | 567 | 702 | 1971 | 296 | 674 | 970 |
| FRM04 | FRM | 458 | 492 | 1442 | 209 | 540 | 749 |
| FRM05 | FRM | 340 | 489 | 1318 | 223 | 410 | 633 |
| FRM06 | FRM | 486 | 599 | 1684 | 275 | 594 | 869 |
| FRM07 | FRM | 421 | 475 | 1371 | 237 | 509 | 746 |
| FRM08 | FRM | 577 | 723 | 2023 | 311 | 707 | 1018 |
| FRM09 | FRM | 546 | 591 | 1728 | 229 | 661 | 890 |
| FRM10 | FRM | 554 | 663 | 1880 | 287 | 649 | 936 |
| KRU01 | KRU | 611 | 740 | 2091 | 303 | 726 | 1029 |
| KRU02 | KRU | 596 | 719 | 2034 | 313 | 725 | 1038 |
| KRU03 | KRU | 656 | 706 | 2068 | 309 | 802 | 1111 |
| KRU04 | KRU | 663 | 716 | 2095 | 294 | 801 | 1095 |
| KRU05 | KRU | 618 | 723 | 2064 | 304 | 741 | 1045 |
| KRU06 | KRU | 591 | 739 | 2069 | 325 | 701 | 1026 |
| KRU07 | KRU | 722 | 706 | 2134 | 267 | 857 | 1124 |
| KRU08 | KRU | 588 | 734 | 2056 | 264 | 718 | 982 |
| KRU09 | KRU | 641 | 726 | 2093 | 295 | 768 | 1063 |
| KRU10 | KRU | 633 | 716 | 2065 | 299 | 755 | 1054 |
| Kenya | MTP | 0 | 953 | 1906 | 0 | 0 | 0 |
| MTP01 | MTP | 532 | 507 | 1546 | 179 | 637 | 816 |
| MTP02 | MTP | 582 | 597 | 1776 | 234 | 685 | 919 |
| MTP03 | MTP | 544 | 564 | 1672 | 211 | 678 | 889 |
| MTP04 | MTP | 606 | 674 | 1954 | 253 | 733 | 986 |
| MTP05 | MTP | 551 | 613 | 1777 | 233 | 660 | 893 |
| MTP06 | MTP | 631 | 603 | 1837 | 200 | 754 | 954 |
| MTP07 | MTP | 613 | 554 | 1721 | 217 | 737 | 954 |
| MTP08 | MTP | 524 | 505 | 1534 | 190 | 628 | 818 |
| MTP09 | MTP | 580 | 525 | 1630 | 189 | 694 | 883 |
| MTP10 | MTP | 523 | 518 | 1559 | 188 | 619 | 807 |

**Table S5.** Genes potentially under positive selection in three wild dog populations (managed metapopulation - MTP, free-roaming population – FRM, and Kruger National Park – KNP) in South Africa and Zimbabwe, using XPEHH values. Genes overlap with windows with xpehh>2 or <-2, and FST in 95 percentile.

| FRM | KNP | MTP-FRM | MTP-KNP |
| --- | --- | --- | --- |
| ALDH2  ANKRD26  ASB4  CHKB  COQ2  FAM81B  GPR171  MED12L  METAP2  P2RY14  PCSK1  PFN2  RNF13  RPL31  SEC24A | ARL9  AZI2  CDV3  CHD1L  CMC1  DHFR  GAPDH  NABP1  PCLAF  RPL10  RPL31  Sec31a  SRP72  TMTC1  TRIP4  ZCWPW2 | HLF  NAAA  SDAD1  SEMA5A | BAG2  EHF  JAK1  RAVER2  SAMD12  SORBS1 |

**Table S6**. Most significantly enriched GO terms in the tests were the root terms, e.g., molecular function (GO:0003674), cellular process (GO:0009987), biological_process (GO:0008150) in Webgestalt.com.

| **Geneontology** | **Gene Set** | **Description** | **Size** | **Expect** | **Ratio** | **P** |
| --- | --- | --- | --- | --- | --- | --- |
| Biological process | GO:0035914 | skeletal muscle cell differentiation | 25 | 0.01 | 91.60 | 0.01 |
| Biological process | GO:0006357 | regulation of transcription by RNA polymerase II | 490 | 0.21 | 9.35 | 0.01 |
| Biological process | GO:0006366 | transcription by RNA polymerase II | 520 | 0.23 | 8.81 | 0.01 |
| Biological process | GO:0007519 | skeletal muscle tissue development | 41 | 0.02 | 55.85 | 0.02 |
| Biological process | GO:0060538 | skeletal muscle organ development | 42 | 0.02 | 54.52 | 0.02 |
| Biological process | GO:0006355 | regulation of DNA-templated transcription | 702 | 0.31 | 6.52 | 0.02 |
| Biological process | GO:2001141 | regulation of RNA biosynthetic process | 704 | 0.31 | 6.51 | 0.02 |
| Biological process | GO:0006351 | DNA-templated transcription | 755 | 0.33 | 6.07 | 0.03 |
| Biological process | GO:0032774 | RNA biosynthetic process | 761 | 0.33 | 6.02 | 0.03 |
| Biological process | GO:0051252 | regulation of RNA metabolic process | 773 | 0.34 | 5.93 | 0.03 |
| Cellular conponent | GO:0090575 | RNA polymerase II transcription regulator complex | 69 | 0.03 | 35.34 | 0.03 |
| Cellular conponent | GO:0005667 | transcription regulator complex | 130 | 0.05 | 18.76 | 0.05 |
| Cellular conponent | GO:0140513 | nuclear protein-containing complex | 351 | 0.14 | 6.95 | 0.14 |
| Cellular conponent | GO:0005634 | nucleus | 1866 | 0.77 | 2.61 | 0.15 |
| Cellular conponent | GO:0005654 | nucleoplasm | 926 | 0.38 | 2.63 | 0.34 |
| Cellular conponent | GO:0031981 | nuclear lumen | 1046 | 0.43 | 2.33 | 0.38 |
| Cellular conponent | GO:0031974 | membrane-enclosed lumen | 1178 | 0.48 | 2.07 | 0.42 |
| Cellular conponent | GO:0043233 | organelle lumen | 1178 | 0.48 | 2.07 | 0.42 |
| Cellular conponent | GO:0070013 | intracellular organelle lumen | 1178 | 0.48 | 2.07 | 0.42 |
| Cellular conponent | GO:0032991 | protein-containing complex | 1509 | 0.62 | 1.62 | 0.52 |
| Molecular function | GO:0060590 | ATPase regulator activity | 11 | 0.01 | 132.18 | 0.01 |
| Molecular function | GO:0003700 | DNA-binding transcription factor activity | 271 | 0.19 | 10.73 | 0.01 |
| Molecular function | GO:0043565 | sequence-specific DNA binding | 339 | 0.23 | 8.58 | 0.02 |
| Molecular function | GO:0140110 | transcription regulator activity | 359 | 0.25 | 8.10 | 0.02 |
| Molecular function | GO:0051087 | protein-folding chaperone binding | 34 | 0.02 | 42.77 | 0.02 |
| Molecular function | GO:0003677 | DNA binding | 560 | 0.39 | 5.19 | 0.05 |
| Molecular function | GO:0097159 | organic cyclic compound binding | 1564 | 1.08 | 2.79 | 0.05 |
| Molecular function | GO:0001228 | DNA-binding transcription activator activity, RNA polymerase II-specific | 136 | 0.09 | 10.69 | 0.09 |
| Molecular function | GO:0001216 | DNA-binding transcription activator activity | 138 | 0.09 | 10.54 | 0.09 |
| Molecular function | GO:0003676 | nucleic acid binding | 909 | 0.63 | 3.20 | 0.11 |
